# A phylogenetic backbone for Bivalvia: an RNA-seq approach

**DOI:** 10.1098/rspb.2014.2332

**Published:** 2015-02-22

**Authors:** Vanessa L. González, Sónia C. S. Andrade, Rüdiger Bieler, Timothy M. Collins, Casey W. Dunn, Paula M. Mikkelsen, John D. Taylor, Gonzalo Giribet

**Affiliations:** 1 Museum of Comparative Zoology, Department of Organismic and Evolutionary Biology, Harvard University, Cambridge, MA 02138, USA; 2 Integrative Research Center, Field Museum of Natural History, Chicago, IL 60605, USA; 3 Department of Biological Sciences, Florida International University, Miami, FL 33199, USA; 4 Department of Ecology and Evolutionary Biology, Brown University, Providence, RI 02912, USA; 5 Paleontological Research Institution and Department of Earth and Atmospheric Sciences, Cornell University, Ithaca, NY 14850, USA; 6 Department of Life Sciences, The Natural History Museum, London SW7 5BD, UK

**Keywords:** phylogenomics, mollusca, bivalves, phylogenetics

## Abstract

Bivalves are an ancient and ubiquitous group of aquatic invertebrates with an estimated 10 000–20 000 living species. They are economically significant as a human food source, and ecologically important given their biomass and effects on communities. Their phylogenetic relationships have been studied for decades, and their unparalleled fossil record extends from the Cambrian to the Recent. Nevertheless, a robustly supported phylogeny of the deepest nodes, needed to fully exploit the bivalves as a model for testing macroevolutionary theories, is lacking. Here, we present the first phylogenomic approach for this important group of molluscs, including novel transcriptomic data for 31 bivalves obtained through an RNA-seq approach, and analyse these data with published genomes and transcriptomes of other bivalves plus outgroups. Our results provide a well-resolved, robust phylogenetic backbone for Bivalvia with all major lineages delineated, addressing long-standing questions about the monophyly of Protobranchia and Heterodonta, and resolving the position of particular groups such as Palaeoheterodonta, Archiheterodonta and Anomalodesmata. This now fully resolved backbone demonstrates that genomic approaches using hundreds of genes are feasible for resolving phylogenetic questions in bivalves and other animals.

## 1. Introduction

Among the most important groups of invertebrates are bivalves, a clade of molluscs of extraordinary impact on human endeavours, even in the biomedical field [1,2]. For example, bivalves are a source of animal protein for humans, and major commercial fisheries have long existed worldwide. The world production of bivalves (i.e. oysters, clams, cockles, scallops and mussels) has been steadily increasing since the 1990s to reach 13.6 million metric tonnes (mt) in 2005, comprising about 2.3% of the total world export of fisheries products [3]. Ecologically, owing to their filter-feeding habits, bivalves are major players in coastal ecosystems and reefs, and they constitute one of the dominant groups of macrofauna in the deep sea [4]. It is thus not surprising that many scholars have tried to understand bivalve relationships, using shell morphology and anatomy [5–13], fossils [14–17], and, more recently, molecular sequence data [12,13,18–21]. The most recent of these studies incorporates novel morphological and molecular sequence data from up to nine molecular markers [13], and largely complements prior studies. This later study agrees with prior ones on many key aspects of bivalve phylogeny, including monophyly of the crown group Bivalvia, monophyly of the bivalves with enlarged and complex gills (Autobranchia), and the division of Autobranchia into the clades Pteriomorphia, Palaeoheterodonta and Heterodonta. The clade Heteroconchia (consisting of Palaeoheterodonta, Archiheterodonta and Euheterodonta) is likewise broadly supported in recent molecular analyses [13]. However, recent molecular data based on mitochondrial genes [21–24] have proposed relationships that are at odds with previously published work based on ribosomal genes and morphology, and with more recent phylogenetic work based on nuclear genes [20].

This increasing resolution of bivalve relationships (excepting the mitochondrial studies) is certainly encouraging [13], but several key questions remain debated. One of these is the monophyly of Protobranchia, a group of bivalves with primitive ctenidia, comprising many deep-sea species, whose relationships were recently reviewed [25]. Although traditionally considered one of the subclasses of bivalves, several molecular analyses have found paraphyly of protobranchs with respect to Autobranchia (see a summary of hypotheses in [25]). Monophyly of its three main groups (Solemyida, Nuculida and Nuculanida) was, however, recently supported in a large analysis using a phylogenomic approach [26]. Another recalcitrant issue concerns the relationships among the heteroconchian lineages, Palaeoheterodonta, Archiheterodonta and Euheterodonta. The traditional view places Palaeoheterodonta as sister group to Heterodonta, composed of Archiheterodonta and Euheterodonta [11,12]. However, molecular analyses have also supported a divergence of Archiheterodonta prior to the split of Palaeoheterodonta and Euheterodonta [13], or even a clade composed of Archiheterodonta and Palaeoheterodonta [8,20]. Finally, although the monophyly of Pteriomorphia and Euheterodonta, respectively, is largely undisputed, the internal relationships of both groups remain poorly supported, despite considerable phylogenetic effort for pteriomorphians [27–29] and heterodonts [30–32]. Resolving these relationships is key for further evolutionary and ecological studies using bivalves as models, including dating and inference of the evolution of lineages through time, to study extinction and diversification patterns, and for using them as models for biogeography.

## 2. Material and methods

### (a) Taxon sampling

Transcriptome data were obtained for 40 molluscan taxa, including 31 newly sequenced bivalve transcriptomes that had been selected based on prior studies [13,20,25] to maximize the diversity of living bivalve lineages (electronic supplementary material, table S1). Full genome data were included for the gastropod *Lottia gigantea* [33] and for the pteriomorphian *Pinctada fucata* [34]. All six major bivalve lineages were represented by at least two species: Protobranchia (3), Pteriomorphia (6), Palaeoheterodonta (3), Archiheterodonta (3), Anomalodesmata (2) and Imparidentia (17). Tissues were preserved in three ways for RNA work: (i) flash-frozen in liquid nitrogen and immediately stored at −80°C; (ii) immersed in at least 10 volumes of RNA*later* (Ambion) and frozen at −80°C or −20°C; (iii) transferred directly into Trizol reagent (Invitrogen, Carlsbad, CA) and immediately stored at −80°C.

### (b) RNA isolation and mRNA extraction

Total RNA was extracted using standard protocols. Following mRNA purification, samples were treated with Ambion turbo DNA-free DNase to remove residual genomic and rRNA contaminants. Quantity and quality (purity and integrity) of mRNA were assessed using a NanoDrop ND-1000 UV spectrophotometer (ThermoFisher Scientific, Wilmington, MA). Quantity of mRNA was also assessed by qubit fluorometer (Invitrogen) and using an Agilent Bioanalyzer 2100 system with the ‘mRNA pico series II’ assay (Agilent Technologies, Santa Clara, CA).

### (c) Next-generation sequencing

Next-generation sequencing (NGS) was carried out using the Illumina HiSeq 2000 platform (Illumina Inc., San Diego, CA) at the FAS Center for Systems Biology at Harvard University. After mRNA extraction, SuperScript III reverse transcriptase was used to amplify cDNA gene products. cDNA was ligated to Illumina TruSeq RNA multiplex adaptor sequences using the TruSeq RNA sample prep kit (Illumina). No more than six adaptors were used per individual multiplexed sequencing run. Size-selected cDNA fragments of 250–350 bp excised from a 2% agarose gel were amplified using Illumina PCR primers for paired-end reads (Illumina), and 15 cycles of the PCR programme comprising 98°C for 30 s, 98°C for 10 s, 65°C for 30 s and 72°C for 30 s, followed by an extension step of 5 min at 72°C.

The concentration of the cDNA libraries was measured with the qubit dsDNA high-sensitivity (HS) assay kit using the qubit fluoremeter (Invitrogen). The quality of the library and size selection was checked using the HS DNA assay in a DNA chip for Agilent Bioanalyzer 2100 (Agilent Technologies). Concentrations of sequencing runs were normalized based on final concentrations of fragmented cDNA. Illumina sequenced paired-end reads were 101 bp. Raw read sequence data have been deposited in NCBI's sequence read archive (SRA) database: BioProject PRJNA242872.

### (d) Data processing

Illumina HiSeq 2000 pair-end reads obtained ranged from 7 867 647 to 51 464 822 per taxon. Data (unprocessed reads) obtained from the SRA database (http://www.ncbi.nlm.nih.gov/sra) were downloaded as raw reads and processed in the same manner as the newly generated transcriptome data. Quality of reads was visualized with FastQC (http://www.bioinformatics.bbsrc.ac.uk/projects/fastqc). Initial removal of low-quality reads and TruSeq multiplex index adaptor sequences (Illumina) was performed with Trim Galore! v. 0.3.1 (http://www.bioinformatics.babraham.ac.uk/projects/trim_galore), setting the quality threshold to minimum Phred score of 30. Illumina TruSeq multiplex adaptor sequences were trimmed, specific to the adaptor used in sequencing with the paired-end data flag. A second round of quality threshold filtering (minimum Phred 35) as well as removal of rRNA sequence contamination was conducted in Agalma v. 0.3.2 using the ‘pre-assemble’ pipeline [35].

### (e) *De novo* assembly

Quality-filtered and sanitized high-quality reads (electronic supplementary material, table S1) were assembled with the Trinity de novo Assembler (release 13 07 2011) with 100 GB of memory and a path reinforcement distance of 50 [36]. The number of contigs, the mean contig length, the N50 and the maximum contig length were reported for each *de novo* assembly (electronic supplementary material, table S1). Contigs were mapped against the Swissprot database using the blastx program of the BLAST suite, and the number of contigs returning blast hits was quantified (electronic supplementary material, table S1). All nucleotide sequences were translated with Transdecoder using default parameters [37]. Subsequent peptide translations were filtered for redundancy and uniqueness using CD-Hit v. 4.6.1 under default parameters, and a 95% similarity threshold [38]. Genome data from *Lottia gigantea* and *Pinctada fucata* were incorporated using predicted peptide sequences obtained from public sources. Predicted peptides were further processed, selecting only one peptide per putative unigene, by choosing the longest isoform (i.e. longest ORF) per Trinity subcomponent using a Python script.

### (f) Orthology assignment and matrix construction

Orthology assessment was conducted using OMA standalone v. 0.99t [39,40], on 64 CPUs of a cluster at Harvard University, FAS Research Computing (odyssey.fas.harvard.edu), using default parameters, except with a minimum alignment score of 200, a length tolerance ratio of 0.75 and a minimum sequence length of 100. A total of 68 828 informative putative orthogroups (more than four taxa) were obtained; orthogroups and genes are referred to interchangeably. Resultant gene clusters were aligned with MAFFT [41] prior to concatenation.

**Figure 1.**
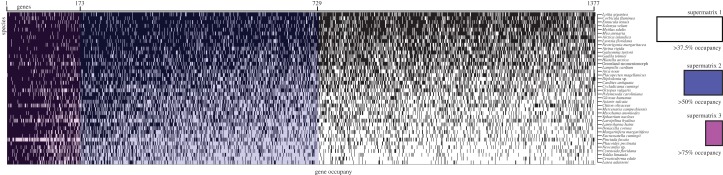
Schematic of the three supermatrices analysed in this study: supermatrix 1 (white: >37.5% gene occupancy, 1377 genes, 46.1% missing data), supermatrix 2 (blue: >50% gene occupancy, 729 genes, 35.4% missing data), supermatrix 3 (pink: >75% gene occupancy, 173 genes, 16% missing data).

We constructed three phylogenetic supermatrices (figure 1) from the translated amino acid sequences. Supermatrices were constructed based on gene occupancy threshold filters—meaning that a gene was selected if found in more than or equal to the established threshold; a 50% threshold would select all genes present in 50% or more of the included taxa. The more than 37.5%, 50% and 75% gene occupancy matrices were then trimmed with Gblocks [42] to cull regions of dubious alignment to be used in downstream phylogenetic reconstructions. Data used in downstream analyses have been deposited in Dryad (http://dx.doi.org/10.5061/dryad.v31ms).

### (g) Phylogenetic and gene tree analyses

Maximum-likelihood tree searches on the three occupancy data matrices were conducted with RAxML v. 7.2.7 [43]. Maximum-likelihood analyses in RAxML specified a model of protein evolution with corrections for a discrete gamma distribution with the LG model [44] to conduct the tree searches, with 100 independent replicates. Bootstrap resampling was conducted for 100 replicates using a rapid bootstrapping algorithm [45] specifying a model of protein evolution with corrections for a discrete gamma distribution using the WAG model [46], and were thereafter mapped onto the optimal tree from the independent searches. Concomitantly, tree searches were conducted for all three data matrices in PhyloBayes MPI v. 1.4e [47] using the site-heterogeneous CAT + GTR model of evolution [48]. Four independent chains were run for 5077–28 310 cycles, and the initial cycles discarded as burn-in were determined for each analysis using the ‘tracecomp’ executable, with convergence assessed using the maximum bipartition discrepancies across chains (maxdiff < 0.3).

In order to quantify gene tree incongruence, visualizations of the dominant bipartitions among individual loci (based on the ML gene tree topologies) were conducted by constructing supernetworks using the SuperQ method selecting the ‘balanced’ edge-weight with ‘Gurobi’ optimization function, and applying no filter [49]. This methodology decomposes all gene trees into quartets to build supernetworks where edge lengths correspond to quartet frequencies. Resulting supernetworks were visualized in SplitsTree v. 4.13.1 [50]. Supernetworks were inferred for all three datasets: (i) 1377 loci, (ii) 729 loci and (iii) 173 loci.

## 3. Results and discussion

### (a) A phylogenomic dataset for bivalves

Phylogenomic analyses to investigate animal relationships have flourished in the past decade [51–53], and a series of tools, driven by NGS technologies, have increased dramatically the size of datasets applied to phylogenetic questions, including molluscan relationships [26,54–56]. It is within this framework of combining NGS technologies and phylogenomic techniques that we decided to re-investigate the last major unresolved nodes in bivalve phylogeny and address the specific questions of protobranch monophyly, the interrelationships of the heteroconchian lineages and the internal relationships of Imparidentia—a clade composed of Myoida and most of the former Veneroida [13]. We thus generated a new dataset, entirely based on transcriptome and genome data (electronic supplementary material, table S1), and constructed multiple matrices from 173 to 1377 genes, and with a gene occupancy ranging between more than 37.5% and more than 75% (see Material and methods; figure 1 and table 1) to investigate these previously unresolved nodes of the bivalve tree of life. These represent the largest (in number of genes; up to 1377) and most complete (in terms of gene occupancy; more than 84%) datasets applied to resolving questions in molluscan relationships.

**Table 1.** Summary of support values for phylogenetic relationships of major bivalve lineages for all six analyses of the three supermatrices.

	matrix occupancy					
	>37.5%		>50%		>75%	
number of loci	1377		729		173	
alignment size (AA)	231 823		117 190		27 732	
missing data (%)	46.6		35.4		16.1	
monophyly of (BS/PP)	RAxML	PhyloBayes	RAxML	PhyloBayes	RAxML	PhyloBayes
Bivalvia	100	1.0	100	1.0	100	1.0
Autobranchia	100	1.0	100	1.0	100	1.0
Heteroconchia	100	1.0	100	1.0	100	1.0
Heterodonta	100	1.0	100	1.0	100	1.0
Euheterodonta	100	1.0	100	1.0	100	1.0
Protobranchia	100	1.0	100	1.0	96	0.99
Pteriomorpha	100	1.0	100	1.0	100	1.0
Palaeoheterodonta	100	1.0	100	1.0	100	1.0
Archiheterodonta	100	1.0	100	1.0	100	1.0
Anomalodesmata	100	1.0	100	1.0	100	1.0
Imparidentia	100	1.0	100	1.0	100	1.0

Concatenated supermatrices were compiled using a threshold of percentage gene occupancy. The number of genes present in each supermatrix varied by taxon, with the most genes being represented in two protobranch taxa, *Ennucula tenuis* and *Solemya velum* (figure 1). All three supermatrices contain data for all of the 40 species included in the study, though taxa varied in gene representation (electronic supplementary material, table S2). Taxa with the fewest parsed characters were *Cerastoderma edule* and *Yoldia limatula*, with only 25.2% and 23.6% of the total genes present in the largest supermatrix.

### (b) Bivalve relationships resolved

**Figure 2.**
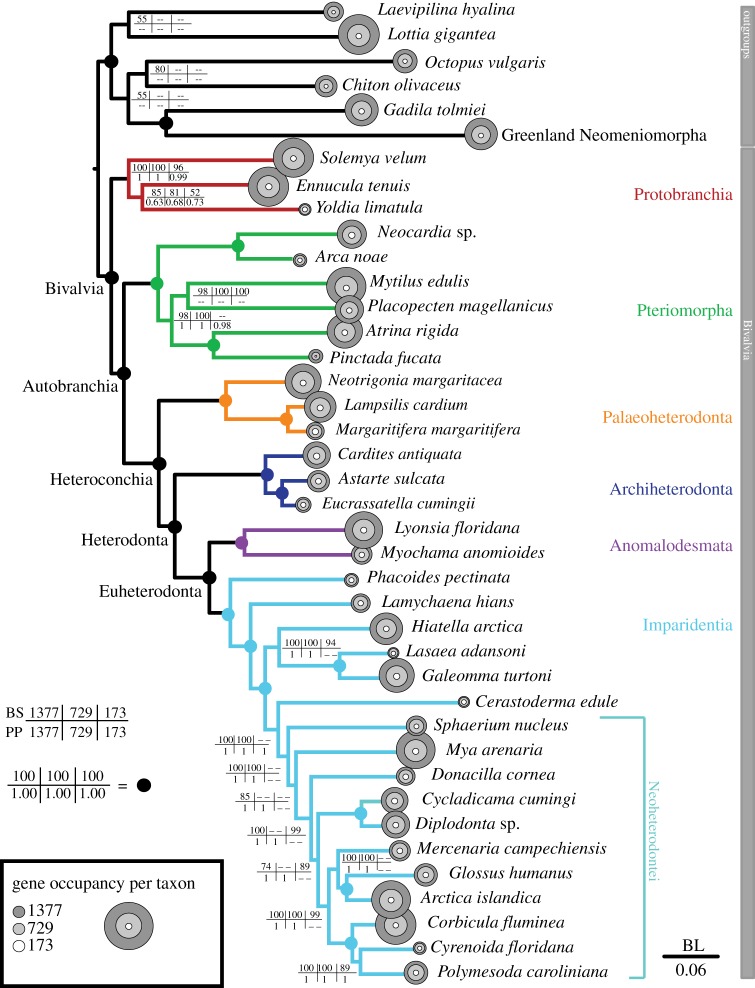
Phylogenetic hypothesis based on the analysis of supermatrix 1 (>37.5% occupancy; 1377 genes; 231 823 amino acids; 46.6% missing data) under the ML method and PROTGAMMALG model. The main bivalve lineages are illustrated in different colours and all taxonomic names used in the text are indicated at different nodes. Circles in nodes indicate maximum support for all ML and PhyloBayes analyses; otherwise, the bootstrap support values and posterior probabilities are indicated on each node. Circles on tips indicate the number of genes represented for each terminal for the three data matrices analysed. Likelihood scores for the three supermatrices are: >37.5% −ln*L* = 3 858 777.48; >50% −ln*L* = 2 099 078.28; >75% −ln*L* = 517 419.93.

Transcriptomic-scale analyses of the three datasets (173 genes, 16% missing data; 729 genes, 35.4% missing data; to 1377 genes, 46.1% missing data) resulted in robust resolution and stable relationships of all major bivalve lineages (see table 1), corroborating some traditional results based on non-numerical cladistic analyses of palaeontological and morphological data [9,11,14] and recent phylogenetic analyses of bivalves. This constitutes the most comprehensive phylogenetic dataset to date for inferring deep relationships within Bivalvia, resulting in robust support in all analyses for higher-level taxonomic relationships for Bivalvia and its major lineages Autobranchia, Heteroconchia and Heterodonta (figure 2; electronic supplementary material, figure S1).

**Figure 3.**
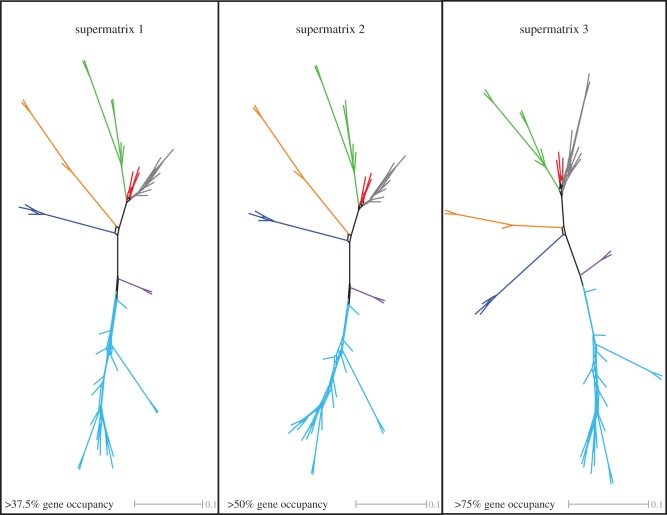
Supernetwork representation of quartets derived from individual ML gene trees, for three different supermatrices. Phylogenetic conflict is represented by reticulations. Edge lengths correspond to quartet frequencies.

All phylogenetic analyses, irrespective of the data matrix or the model of sequence evolution analysed, recovered highly congruent topologies throughout Bivalvia, including all currently recognized bivalve subclasses and their major divisions (figure 2; table 1; electronic supplementary material, figure S1)—the deep backbone of the bivalve tree. Analysis of the three datasets recovered monophyly of Protobranchia, irrespective of the method or model of protein evolution used, but the smallest matrix did not obtain maximum support for Protobranchia (96% bootstrap support; posterior probability = 0.99). Likewise, the supernetwork representation of the gene trees, designed to demonstrate putative gene conflict, shows a topology compatible with that of the phylogenetic trees, although the edge separating the outgroups, Protobranchia (red) and Pteriomorphia (green), is short in this case (figure 3), therefore pointing at some sort of discrepancy between some individual gene trees and the concatenated datasets.

A major controversy in molecular studies of bivalve relationships has been the relationships between three well-established clades within Heteroconchia: Palaeoheterodonta, Archiheterodonta and Euheterodonta. Archiheterodonta and Euheterodonta have been traditionally grouped in the subclass Heterodonta. Palaeoheterodonta includes two main lineages: the diverse freshwater mussels (of conservation importance) and the marine-living fossil *Neotrigonia* [57], only known from Australian waters [58]. Archiheterodonta includes three families of primitive, exclusively marine asiphonate species [59]. Euheterodonta divides into Anomalodesmata—a group with unusual morphology prominent in the deep-sea and including the only lineage of carnivorous bivalves [60]—and Imparidentia [13], the latter including some of the best-known bivalves and most of the commercial species (excluding mussels, oysters and their relatives, which are members of Pteriomorphia). A recent debate in the literature involved the resolution of these three heteroconchian clades, with most traditional studies supporting the palaeontological view of an early branching of Palaeoheterodonta, but some more recent molecular studies supporting either an early split of Archiheterodonta, or a sister group relationship of Palaeoheterodonta and Archiheterodonta [13,20]. Our phylogenomic analyses recover the traditional monophyly of Heterodonta (Archiheterodonta as sister group to Euheterodonta), and the DNA sequence-based division of Euheterodonta into Anomalodesmata and Imparidentia, closing decades of debate in the bivalve literature. Gene tree analyses identified some conflict here, but the edge separating Palaeoheterodonta (orange) from Archiheterodonta + Euheterodonta is longer than that placing Archiheterodonta (navy blue) with Palaeoheterodonta (figure 3).

Internal resolution of Imparidentia has been difficult to clarify using traditional Sanger-based markers and morphology [12,13,31,32], but many relationships find full support in all our phylogenomic datasets, whether based on concatenation or on gene trees. Salient resolved nodes include the sister group relationship of *Lamychaena hians* (Gastrochaenidae) to the non-lucinid Imparidentia, one of the most problematic families to place in bivalve phylogenies [13] owing to the modifications imposed by their hard-substratum boring habits. The relationship of *Arctica islandica* to *Glossus humanus* also receives maximal support herein, as does the monophyly of Ungulinoidea (*Cycladicama cumingi* and *Diplodonta* sp.). One of the best-supported imparidentian clades is Cyrenoidea (formerly Corbiculoidea), a group here represented by *Corbicula fluminea*, *Cyrenoida floridana* and *Polymesoda caroliniana*. Cyrenoidea, a group of bivalves largely adapted to low-salinity environments, had already found support in previous molecular analyses [13,20,61], a finding here corroborated, and one that conflicts with many traditional classifications of bivalves. The position of all other Imparidentia is largely congruent with previous hypotheses [13,20], and finds absolute support (100% bootstrap and 1.00 posterior probability) in at least some of the analyses, especially for the largest datasets. These include clades such as Neoheterodontei [32], which receives maximal support from the analyses of the two largest matrices, and many of its subclades (figure 3).

### (c) Remaining gaps in our understanding of bivalve relationships

Monophyly of Protobranchia was supported in previous molluscan phylogenomic analyses [26,55], and in recent Sanger-based molecular analyses of bivalves [13,20]. The latest molecular analysis of protobranch relationships using traditional molecular markers found it difficult to resolve the internal relationships of the major protobranch lineages (Solemyida, Nuculida and Nuculanida), but mostly retrieved a sister group relationship of Nuculida and Nuculanida, with Solemyida as their external clade [25]. This relationship is evident in all analyses for the three largest matrices studied here, in which Nuculida and Nuculanida form a clade. However, support for this relationship is low (figure 2), and gene conflict is strong in this part of the tree (figure 3, red), although this could be owing to the poor library quality for *Yoldia limatula* (figures 1 and 2). Expanded taxon sampling may help to definitively resolve the internal relationships of the earliest-branching bivalve clade, but our approach nevertheless resolves the monophyly of the clade with high support.

The relationships among some imparidentian families still remain unclear, because this study was designed to test the deep divergences among the main lineages of bivalves, and not particular imparidentian families. This phylogenomic approach, however, resolves several unsettled aspects of heterodont phylogeny, including the position of the previously difficult to place Gastrochaenidae and Cardiidae, and supporting several groups, including Neoheterodontei, bringing great promise on how to investigate relationships among the bivalve families of higher branches. Our approach thus sets the stage for testing the phylogenetic placement of unstable families such as Thyasiridae and Chamidae, among others. Future attention should now be directed to broadening the sampling within Pteriomorphia and Imparidentia.

### (d) A resolved bivalve tree of life?

Whereas some discordance of traditional relationships of Bivalvia has persisted in the literature, especially between hypotheses based on morphological, palaeontological and molecular datasets, here we provide a robust resolution of deep bivalve lineages. Our transcriptomic data corroborate many traditional taxonomic groupings based on disparate sources of data, from fossils to molecules, and highlight that historical discordance among bivalve classification is often not due to the choice of palaeontological versus neontological, or molecular versus morphological sets of characters proper, but contingent on basing taxonomic decisions on single or a few preferred character systems. For example, palaeontologists favoured an early split of Palaeoheterodonta and Heterodonta, and an early divergence of Archiheterodonta within Heterodonta [14], whereas some recent molecular analyses challenged this arrangement [13,20]. On the other hand, neither palaeontologists nor morphologists have placed Anomalodesmata nested within Euheterodonta, a result that is prevalent in nearly all molecular analyses. Our enlarged molecular datasets corroborate the latter molecular-based position of Anomalodesmata, but support the traditional palaeontological proposal for the early divergence of Heteroconchia.

A resolved bivalve tree of life allows us to address subsequent evolutionary questions for which bivalves are ideal study subjects owing to their ubiquity in all water systems, latitudes and depths. For example, protobranchs have been used as models to study extinction and diversification because they preserve the signature of the end-Permian mass extinction [25]. Owing to their rich and old fossil record, bivalves have been used in large-scale macroevolutionary studies [62–64]. By combining an exemplary fossil record, extensive morphological knowledge, and the available genomic and transcriptomic (mostly provided here) resources now covering all major bivalve clades, we can not only provide a solid phylogenetic framework for bivalves but also begin to explore many other key aspects of their evolution.

### (e) Bivalve phylogenomics

In the beginning, phylogenomic approaches in animals were applied to deep evolutionary questions to resolve, for example, relationships among the animal phyla [51,52], but costs were prohibitive for attempting more focused taxonomic studies. The past few years have seen an explosion of phylogenomic studies now focusing on many different animal phyla or in sections of these phyla [26,54,55], but many of these still added one or a few species to pre-existing datasets (often incomplete or mixing genomes, transcriptomes and ESTs), or were relatively small. In fact, in our tree, we can easily spot the first libraries sequenced for this study, as they include the taxa with the smallest gene representation (figure 2), highlighting the rapid improvement of RNA-seq techniques even at very short time scales.

Another particularity of the bivalve tree is the apparent lack of major conflict typically shown in many other recent phylogenomic datasets that appear to be more sensitive to missing data, gene selection and effects of heterotachy, compositional biases and other confounding factors in phylogenomic reconstruction [65–67]. This made our study relatively straightforward, as we were able to show that neither missing data nor matrix size, nor the different evolutionary models taking into account site heterogeneity, identified any major conflicts. To a large extent, the individual gene trees for all matrices also showed congruence with the concatenated datasets, supporting the major finding of a well-resolved backbone for Bivalvia. This is, however, not the case for the outgroup taxa, which are poorly resolved and show inconsistent results among analyses, although one clade, composed of Neomeniomorpha and Scaphopoda, received full support in all analyses (figure 2; electronic supplementary material, figure S1). The latter clade is at odds with any previous relationship proposed for such taxa, and Scaphopoda tends to be unstable in other published phylogenomic trees [26,54,56]. This probably results from the absence of Chaetodermomorpha in the datasets, allowing an attraction of the long-branched Neomeniomorpha and the unstable Scaphopoda.

To date, few studies have been published with the amount of novel data presented here (31 new transcriptomes) for an analysis below the phylum level (but see our gastropod study [56]), yet such an effort is now perfectly feasible. At this rate, if tissues become available, sequencing hundreds of bivalves in this fashion should be an achievable community effort. We hope that our tree (and publicly available associated data) serves as a catalyst for continuing to advance knowledge of the bivalve evolutionary chronicle.

## Supplementary Material

Supplementary Figure

## Supplementary Material

Supplementary Table 1

## Supplementary Material

Supplementary Table 2
