# Supplementary figures and images for: A phylogenetic backbone for Bivalvia: an RNA-seq approach
Source: Proc Biol Sci. 2015 Feb 22;282(1801):20142332. doi: 10.1098/rspb.2014.2332 (PMC4308999; doi:10.1098/rspb.2014.2332)

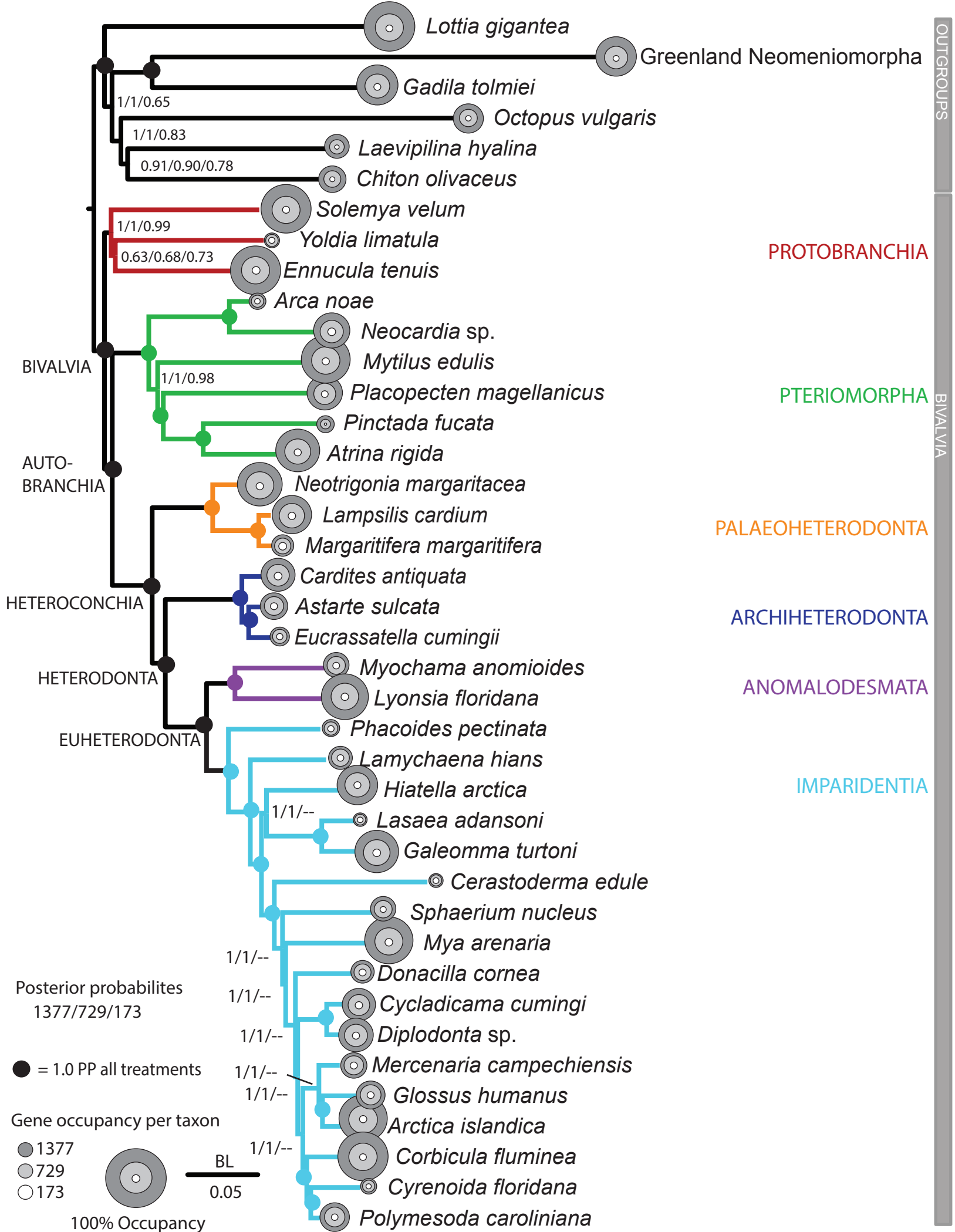

Supplement: Supplementary Figure [file rspb20142332supp1.pdf]
