# Supplementary material for: A phylogenetic backbone for Bivalvia: an RNA-seq approach
Source: Proc Biol Sci. 2015 Feb 22;282(1801):20142332. doi: 10.1098/rspb.2014.2332 (PMC4308999; doi:10.1098/rspb.2014.2332)
Supplement: Supplementary Table 1 [file rspb20142332supp2.docx]

**Suppl. Table 1.** List of species sampled with voucher numbers and read pair information from subsequent data processing, included in the phylogenomic analyses. BivAToL voucher numbers refer to FMNH specimens. Bold SRA numbers refer to new transcriptomes from this study. Taxonomy mostly follows Bieler *et al.* (68).

| Taxa | SRA Accession | Voucher Number | Post Filtering HiQual Reads | Assembled Contigs (NT) | Average Contig length | N50 | Longest Contig | SwissProt Hits | Unique Translated Contigs (AA) |
| --- | --- | --- | --- | --- | --- | --- | --- | --- | --- |
| **PROTOBRANCHA** |  |  |  |  |  |  |  |  |  |
| *Ennucula tenuis* (Montagu, 1808) | SRX091980/SRR331123 | -- | 28,450,918 | 172,251 | 785 | 1,289 | 28,747 | 31,770 | 22,697 |
| *Solemya velum* Say, 1822 | SRX091478/SRR330465 | -- | 22,761,869 | 98,156 | 1,215 | 2,646 | 32,991 | 24,520 | 16,007 |
| *Yoldia limatula*  (Say, 1831) | **SRX687760/SRS690222** | BivAToL 19.1a/3a | 26,199,671 | 15,867 | 533 | 521 | 13,885 | 5,950 | 3,671 |
| **PTERIOMORPHA** |  |  |  |  |  |  |  |  |  |
| *Arca noae* Linnaeus, 1758 | **SRX687762/SRS690228** | BivAToL 116.1a | 40,199,838 | 116,424 | 437 | 471 | 14,750 | 19,357 | 10,773 |
| *Neocardia* sp. | **SRX701839/SRS701705** | MCZ 378927 | 6,970,286 | 69,366 | 380 | 388 | 8,516 | 10,088 | 8,998 |
| *Atrina rigida* (Lightfoot, 1786) | **SRX687763/SRS690428** | BivAToL 14.1a | 20,116,658 | 85,272 | 644 | 927 | 21,831 | 16,537 | 10,740 |
| *Mytilus edulis* Linnaeus, 1758 | **SRX687765/SRS690430** | MCZ 381397 | 20,427,253 | 80,287 | 612 | 642 | 19,256 | 27,737 | 23,815 |
| *Pinctada fucata* (Gould, 1850) | DRX001100/DRR001602 | -- | -- | -- | -- | -- | -- | 21,002 | 42,042 |
| *Placopecten magellanicus* (Gmelin, 1791) | **SRX687766/SRS690431** | BivAToL 360.1a | 13,721,030 | 30877 | 758 | 913 | 30,116 | 16,354 | 9,736 |
| **PALAEOHETERODONTA** |  |  |  |  |  |  |  |  |  |
| *Lampsilis cardium* Rafinesque, 1820 | **SRX687767/SRS690432** | BivAToL 421.5a | 14,819,846 | 108,039 | 493 | 589 | 13,298 | 13,494 | 10,192 |
| *Margaritifera margaritifera* (Linnaeus, 1758) | **SRX687769/SRS690434** | BivAToL 299.2d | 10,596,660 | 50,736 | 514 | 509 | 13,399 | 8,158 | 7,526 |
| *Neotrigonia margaritacea* (Lamarck, 1804) | **SRX687770/SRS690435** | MCZ 379092 | 22,048,954 | 162,657 | 490 | 549 | 29,210 | 18,261 | 14,988 |
| **ARCHIHETERODONTA** |  |  |  |  |  |  |  |  |  |
| *Astarte sulcata* (da Costa, 1778) | **SRX687759/SRS690427** | MCZ 378853 | 32,670,541 | 76,320 | 451 | 512 | 6,976 | 9,586 | 8,326 |
| *Eucrassatella cumingii*  (A. Adams, 1854) | **SRX687772/SRS690437** | BivAToL 83.1b | 21,838,500 | 102,094 | 395 | 395 | 13,256 | 10,051 | 6,757 |
| *Cardites antiquata* (Linnaeus, 1758) | **SRX687773/SRS690438** | MCZ Spain | 25,712,910 | 113,906 | 487 | 567 | 12478 | 16,237 | 10,798 |
| **ANOMALODESMATA** |  |  |  |  |  |  |  |  |  |
| *Lyonsia floridana* Conrad, 1849 | **SRX687774/SRS690439** | BivAToL 248.1a | 20,343,583 | 92076 | 588 | 838 | 8,775 | 25,319 | 18,084 |
| *Myochama anomioides* Stutchbury, 1830 | **SRX687775/SRS690440** | BivAToL 84.1a | 32,300,919 | 120,487 | 416 | 440 | 16,127 | 12,073 | 8,883 |
| **IMPARIDENTIA** |  |  |  |  |  |  |  |  |  |
| *Arctica islandica* (Linnaeus, 1767) | **SRX687761/SRS690229** | BivAToL 191.3a | 49,579,535 | 161,090 | 529 | 669 | 21,022 | 22,478 | 17,823 |
| *Cerastoderma edule*  (Linnaeus, 1758) | **SRX687776/SRS690441** | BivAToL 21.1a | 32,707,689 | 31,719 | 389 | 401 | 4,604 | 5,294 | 3,815 |
| *Corbicula fluminea* (O.F. Müller, 1774) | **SRX687764/SRS690429** | BivAToL 242.1a | 48,293,353 | 176,007 | 576 | 763 | 27,648 | 30,161 | 21,609 |
| *Cyrenoida floridana* Dall, 1896 | **SRX687777/SRS690442** | BivAToL 27.1a/2a | 26,193,865 | 69,160 | 396 | 420 | 10,756 | 7,360 | 7,451 |
| *Galeomma turtoni* Turton, 1825 | **SRX687768/SRS690433** | MCZ 378975 | 14,494,707 | 92,358 | 475 | 548 | 12,194 | 23,822 | 18,420 |
| *Lasaea adansoni* (Gmelin, 1791) | **SRX687778/SRS690443** | BivAToL 268.4 | 13,598,439 | 210,853 | 336 | 328 | 9,602 | 4,957 | 9,223 |
| *Lamychaena hians* (Gmelin, 1791) | **SRX687779/SRS690444** | BivAToL 289.1b | 37,399,760 | 67,366 | 392 | 400 | 7,972 | 8,670 | 5,990 |
| *Glossus humanus* (Linnaeus, 1758) | **SRX687780/SRS690445** | BivAToL 200.1a | 10,145,130 | 76,149 | 429 | 458 | 8,949 | 11,772 | 8,806 |
| *Hiatella arctica* (Linnaeus, 1767) | **SRX687781/SRS690446** | BivAToL 195.1a | 36,448,617 | 73,557 | 487 | 576 | 13,375 | 15,063 | 10,583 |
| *Phacoides pectinata* (Gmelin, 1791) | **SRX687782/SRS690447** | BivAToL 278.1b | 5,724,985 | 85,866 | 414 | 432 | 12,666 | 6,589 | 3,989 |
| *Diplodonta* sp. | **SRX701840/SRS701706** | MCZ 378957 | 15,607,921 | 104,958 | 420 | 444 | 14,966 | 14,298 | 9,309 |
| *Cycladicama cumingi* (Hanley, 1844) | **SRX687783/SRS690448** | BivAToL 371.2c | 17,256,202 | 77,024 | 438 | 484 | 7,676 | 10,419 | 7,326 |
| *Polymesoda caroliniana* (Bose, 1801) | **SRX687784/SRS690449** | MCZ 381394 | 44,017,251 | 123,646 | 539 | 679 | 12,358 | 14,923 | 7,580 |
| *Donacilla cornea* (Poli, 1791) | **SRX687785/SRS690451** | BivAToL 406.1a | 12,023,230 | 54,705 | 475 | 564 | 9,281 | 8,184 | 7,249 |
| *Mya arenaria* Linnaeus, 1758 | **SRX687786/SRS690452** | MCZ 381391 | 17,884,175 | 98,870 | 617 | 873 | 35,612 | 22,682 | 15,764 |
| *Sphaerium nucleus* (Studer, 1820) | **SRX687787/SRS690453** | BivAToL 194.1a | 38,431,045 | 228,353 | 376 | 386 | 15,741 | 23,452 | 19,027 |
| *Mercenaria campechiensis* (Gmelin, 1791) | **SRX687788/SRS690454** | MCZ 381392 | 16,027,302 | 54,108 | 460 | 520 | 11,917 | 6,947 | 5,173 |
|  |  |  |  |  |  |  |  |  |  |
| **OUTGROUPS** |  |  |  |  |  |  |  |  |  |
| **NEOMENIOMORPHA** |  |  |  |  |  |  |  |  |  |
| Greenland Neomeniomorpha | SRX092156/SRR331902 | -- | 24,109,577 | 69,154 | 783 | 1,008 | 28,982 | 58,972 | 18,135 |
| **CEPHALOPODA** |  |  |  |  |  |  |  |  |  |
| *Octopus vulgaris* Cuvier, 1797 | SRA044948/SRS257947 | -- | 5,009,720 | 63,005 | 711 | 826 | 12,825 | 19,977 | 15,641 |
| **GASTROPODA** |  |  |  |  |  |  |  |  |  |
| *Lottia gigantea* G. B. Sowerby I, 1834 | JGI | -- | -- | -- | -- | -- | -- | 157,638 | 37,056 |
| **SCAPHOPODA** |  |  |  |  |  |  |  |  |  |
| *Gadila tolmiei* (Dall, 1897) | SRX092154/SRR331897 | -- | 37,024,753 | 106,509 | 459 | 522 | 13,484 | 29,462 | 24,410 |
| **POLYPLACOPHORA** |  |  |  |  |  |  |  |  |  |
| *Chiton olivaceus* Spengler, 1797 | SRX205322/SRA061738 | -- | 17,395,660 | 63,253 | 601 | 644 | 11,858 | 29,067 | 29,361 |
| **MONOPLACOPHORA** |  |  |  |  |  |  |  |  |  |
| *Laevipilina hyalina* J. H. McLean, 1979 | SRX091470/SRR330425 | -- | 21,400,869 | 184,307 | 563 | 714 | 29,374 | 49,370 | 18,437 |
