# Supplementary material for: A phylogenetic backbone for Bivalvia: an RNA-seq approach
Source: Proc Biol Sci. 2015 Feb 22;282(1801):20142332. doi: 10.1098/rspb.2014.2332 (PMC4308999; doi:10.1098/rspb.2014.2332)
Supplement: Supplementary Table 2 [file rspb20142332supp3.docx]

**Suppl. Table 2.** List of number and ratio of genes sampled per taxon, for each minimum gene occupancy threshold used for supermatrix construction: >37.5%, >50% and >75 %.

|  | MATRIX OCCUPANCY (NUMBER OF GENES) | | | | | |
| --- | --- | --- | --- | --- | --- | --- |
| Species | >37.5% (1377) | Total (%) | >50% (729) | Total (%) | >75% (173) | Total (%) |
| **PROTOBRANCHA** |  |  |  |  |  |  |
| *Ennucula tenuis* (Montagu, 1808) | 1140 | 82.8% | 638 | 87.5% | 158 | 91.3% |
| *Solemya velum* Say, 1822 | 1138 | 82.6% | 635 | 87.1% | 157 | 90.8% |
| *Yoldia limatula*  (Say, 1831) | 347 | 25.2% | 282 | 38.7% | 138 | 79.8% |
| **PTERIOMORPHA** |  |  |  |  |  |  |
| *Arca noae* Linnaeus, 1758 | 823 | 59.8% | 526 | 72.2% | 152 | 87.9% |
| *Neocardia* sp*.* | 383 | 27.8% | 283 | 38.8% | 123 | 71.1% |
| *Atrina rigida* (Lightfoot, 1786) | 999 | 72.5% | 588 | 80.7% | 158 | 91.3% |
| *Mytilus edulis* Linnaeus, 1758 | 1109 | 80.5% | 642 | 88.1% | 161 | 93.1% |
| *Pinctada fucata* (Gould, 1850) | 398 | 28.9% | 226 | 31.0% | 54 | 31.2% |
| *Placopecten magellanicus* (Gmelin, 1791) | 804 | 58.4% | 498 | 68.3% | 151 | 87.3% |
| **PALEOHETERODONTA** |  |  |  |  |  |  |
| *Lampsilis cardium* Rafinesque, 1820 | 897 | 65.1% | 573 | 78.6% | 165 | 95.4% |
| *Margaritifera margaritifera* (Linnaeus, 1758) | 502 | 36.5% | 369 | 50.6% | 148 | 85.5% |
| *Neotrigonia margaritacea* (Lamarck, 1804) | 1018 | 73.9% | 635 | 87.1% | 163 | 94.2% |
| **ARCHIHETERODONTA** |  |  |  |  |  |  |
| *Astarte sulcata* (da Costa, 1778) | 624 | 45.3% | 438 | 60.1% | 157 | 90.8% |
| *Eucrassatella cumingii*  (A. Adams, 1854) | 440 | 32.0% | 330 | 45.3% | 142 | 82.1% |
| *Cardites antiquata* (Linnaeus, 1758) | 773 | 56.1% | 508 | 69.7% | 154 | 89.0% |
| **IMPARIDENTIA** |  |  |  |  |  |  |
| *Lyonsia floridana* Conrad, 1849 | 1068 | 77.6% | 625 | 85.7% | 166 | 96.0% |
| *Myochama anomioides* Stutchbury, 1830 | 581 | 42.2% | 418 | 57.3% | 150 | 86.7% |
| *Arctica islandica* (Linnaeus, 1767) | 1096 | 79.6% | 650 | 89.2% | 162 | 93.6% |
| *Cerastoderma edule*  (Linnaeus, 1758) | 325 | 23.6% | 257 | 35.3% | 130 | 75.1% |
| *Corbicula fluminea* (O.F. Müller, 1774) | 1142 | 82.9% | 642 | 88.1% | 151 | 87.3% |
| *Cyrenoida floridana* Dall, 1896 | 366 | 26.6% | 276 | 37.9% | 116 | 67.1% |
| *Galeomma turtoni* Turton, 1825 | 990 | 71.9% | 603 | 82.7% | 171 | 98.8% |
| *Lasaea adansoni* (Gmelin, 1791) | 316 | 22.9% | 251 | 34.4% | 127 | 73.4% |
| *Lamychaena hians* (Gmelin, 1791) | 541 | 39.3% | 404 | 55.4% | 148 | 85.5% |
| *Glossus humanus* (Linnaeus, 1758) | 656 | 47.6% | 470 | 64.5% | 154 | 89.0% |
| *Hiatella arctica* (Linnaeus, 1767) | 923 | 67.0% | 567 | 77.8% | 155 | 89.6% |
| *Phacoides pectinata* (Gmelin, 1791) | 398 | 28.9% | 309 | 42.4% | 141 | 81.5% |
| *Diplodonta* sp. | 786 | 57.1% | 521 | 71.5% | 159 | 91.9% |
| *Cycladicama cumingi* (Hanley, 1844) | 756 | 54.9% | 490 | 67.2% | 164 | 94.8% |
| *Polymesoda caroliniana* (Bose, 1801) | 671 | 48.7% | 462 | 63.4% | 147 | 85.0% |
| *Donacilla cornea* (Poli, 1791) | 530 | 38.5% | 387 | 53.1% | 156 | 90.2% |
| *Mya arenaria* Linnaeus, 1758 | 1096 | 79.6% | 636 | 87.2% | 170 | 98.3% |
| *Sphaerium nucleus* (Studer, 1820) | 576 | 41.8% | 407 | 55.8% | 135 | 78.0% |
| *Mercenaria campechiensis* (Gmelin, 1791) | 596 | 43.3% | 443 | 60.8% | 159 | 91.9% |
|  |  |  |  |  |  |  |
| **OUTGROUPS** |  |  |  |  |  |  |
| Greenland Neomeniomorpha | 922 | 67.0% | 530 | 72.7% | 132 | 76.3% |
| *Octopus vulgaris* Cuvier, 1797 | 684 | 49.7% | 405 | 55.6% | 123 | 71.1% |
| *Lottia gigantea* G. B. Sowerby I, 1834 | 1157 | 84.0% | 638 | 87.5% | 153 | 88.4% |
| *Gadila tolmiei* (Dall, 1897) | 938 | 68.1% | 563 | 77.2% | 153 | 88.4% |
| *Chiton olivaceus* Spengler, 1797 | 623 | 45.2% | 386 | 52.9% | 113 | 65.3% |
| *Laevipilina hyalina* J. H. McLean, 1979 | 559 | 40.6% | 334 | 45.8% | 99 | 57.2% |
